# Evaluation of the efficacy of subinguinal antegrade sclerotherapy and subinguinal micro varicocelectomy versus a combined approach on the semen parameters in infertile patients: a randomized prospective comparative study

**DOI:** 10.1186/s12610-026-00314-4

**Published:** 2026-05-19

**Authors:** Amr Elahwany, Sameh Fayek GamalEl Din, Hesham Torad, Islam ELsisi, Abdel Rahman Hashem, Abdelrahman Ahmed Aburahma

**Affiliations:** 1 Department of Andrology and STDs Kasr Al‑Ainy, Sexual medicine and STIs department, Faculty of Medicine, Cairo University, Al‑Saray Street, El Manial, Cairo, 11956 Egypt; 2 Nile Center for IVF, Cairo, Egypt; 3 Department of urology, Kasr Alainy Faculty of medicine, Cairo University, Cairo, Egypt

**Keywords:** subinguinal micro varicocelectomy, subinguinal antegrade sclerotherapy, combined approach, sperm motility, abnormal forms, Micro-Varicolectomie subinguinale, Sclérothérapie antégrade sous-inguinale, Approche combinée, Motilité des spermatozoïdes, Formes anormales

## Abstract

**Background:**

We assessed whether a combined approach of subinguinal antegrade sclerotherapy (SAS) and subinguinal micro varicocelectomy (MVS) can provide effective and safe treatment of varicocele regarding semen quality and recurrence rate compared to each approach individually.

**Results:**

The median count significantly increased at baseline, and after 3 months, 6 months to be 13.0, 35.0, 40.0, respectively. The median motility significantly increased at baseline, and after 3 months, 6 months to be 37.0, 50.0, and 55.0, respectively. Intra-group comparisons showed significant improvement regarding the median total sperm motility among the participants over the same period. Furthermore, a significant improvement in the median progressive motility among the participants over the same period was observed. Remarkably, the median abnormal forms significantly reduced among the 3 groups at baseline, and after 3 months, 6 months, respectively. Highly significant post-operative reductions in the left and right veins diameters were noted in the intra-group comparisons, with subsequent non visualization of the post-operative left and right veins reflux. Moreover, the median operative time for the left-sided procedures in the 3 groups was 29.5 minutes, 20 minutes, 25 minutes, respectively. For bilateral procedures, the mean operative time was significantly longer in group (A) (57.86 ± 5.2 min) compared to groups B and C (36.93 ± 4.3 min; 44.17 ± 3.45 min, respectively). It should be mentioned that the incidence of orchialgia was significantly absent in the combined approach compared to each approach individually.

**Conclusion:**

Subinguinal MSV and SAS resulted in a significant post-operative improvement in the semen parameters. Nevertheless, combined approach should be recommended as it is associated with the lowest incidence of complications. Future studies with larger scales are needed to assert the current results.

## Résumé

**Contexte:**

Nous avons évalué si une approche combinée de sclérothérapie antégrade sous-inguinale (SAS) et de micro-varicolectomie subinguinale (MVS) pouvait offrir un traitement efficace et sûr de la varicocèle en termes de qualité du sperme et de taux de récidive, par rapport à chaque approche individuelle.

**Résultats:**

La valeur médiane de la concentration de spermatozoïdes de départ a significativement augmenté, après 3 mois et 6 mois, pour atteindre respectivement 13,0, 35,0, 40,0. La valeur médiane de la mobilité des spermatozoïdes de départ a significativement augmenté, après 3 mois et 6 mois pour atteindre respectivement 37,0, 50,0, et 55,0. Les comparaisons intra-groupes ont montré une amélioration significative de la valeur médiane de la mobilité totale des spermatozoïdes chez les participants sur la même période. De plus, on a observé une amélioration significative de la valeur médiane de la motilité progressive parmi les participants sur la même période. Fait remarquable, les valeurs médianes des anormales morphologiques des spermatozoïdes ont considérablement diminué parmi les 3 groupes au départ, puis après 3 mois, 6 mois, respectivement. Des réductions post-opératoires très significatives du diamètre des veines gauche et droite ont été observées lors des comparaisons intra-groupes, avec une non-visualisation ultérieure du reflux postopératoire des veines gauche et droite. Par ailleurs, le temps opératoire médian des interventions du côté gauche dans les 3 groupes était respectivement de 29, 20 et 25 minutes. Pour les interventions bilatérales, le temps opératoire moyen était significativement plus long dans le groupe (A) (57,9 ± 5,2 min) comparé aux groupes B et C (respectivement 36,9 ± 4,3 min ; 44,2 ± 3,45 min). Il convient de mentionner que l’incidence de l’orchialgie était significativement absente dans l’approche combinée par rapport à chaque approche individuellement.

**Conclusions:**

La micro-varicolectomie subinguinale (MVS) et la sclérothérapie antégrade sous-inguinale (SAS) ont induit une amélioration significative des paramètres du sperme après l’opération. Néanmoins, une approche combinée doit être recommandée car elle est associée à la plus faible incidence de complications. Des études futures à plus grande échelle sont nécessaires pour affirmer les résultats actuels.

## Introduction

Varicocele (Vx) can be described as abnormal dilation and tortuosity of the internal spermatic veins and pampiniform plexus [1]. The concept of retrograde blood flow through the internal spermatic vein is crucial in the pathogenesis of impaired spermatogenesis caused by Vx [1]. A distinct correlation exists between infertility, varicocele, and testicular growth arrest [1]. Vx is found in 19% to 41% of men with primary infertility and 45% to 80% of men with secondary infertility and is consequently the most prevalent correctable cause of infertility in males [2]. Consistently, Vx is one of the most prevalent surgically correctable causes of male infertility, affecting a considerable proportion of men and contributes to impaired spermatogenesis and semen quality [3, 4]. It is categorized by dilatation & tortuosity of the pampiniform plexus, leading to testicular hyperthermia, hypoxia, & oxidative stress, that negatively influence sperm function [5]. Several treatment modalities have been introduced for Vx management. Subinguinal microsurgical varicocelectomy (MSV) is widely regarded as the gold standard due to its low recurrence and complication rates, though it requires microsurgical expertise & longer operative time [6]. In contrast, antegrade sclerotherapy is minimally invasive, technically straightforward, and associated with faster recovery, but carries a risk of incomplete occlusion or recurrence [7, 8]. Recently, a combined approach incorporating both techniques has been suggested, aiming to merge the precision of microsurgery with the efficacy of sclerotherapy. Early evidence indicates that this combination may offer superior semen quality outcomes compared with either technique individually [9, 10].

Recently, Elahwany et al. (2025) [11] revealed that Fisch, sclerotherapy and MSV showed significant improvement in the semen parameters after 3 months together with sclerotherapy technique being the most effective in improving the postoperative progressive sperm motility percent compared to MSV. To the best of our knowledge, this is the first prospective randomized study that evaluated the efficacy of either subinguinal MSV or SAS separately versus the combined approach in treating infertile patients with primary Vx. Primary outcome of the study included determination of improvement in sperm count, motility, and morphology at baseline and after 3 months, and 6 months, respectively. Secondary outcomes of the study included pregnancy rate, operative time, recurrence rate, complications rate, patient satisfaction and postoperative recovery.

### Patients and methods

**Fig. 1:**
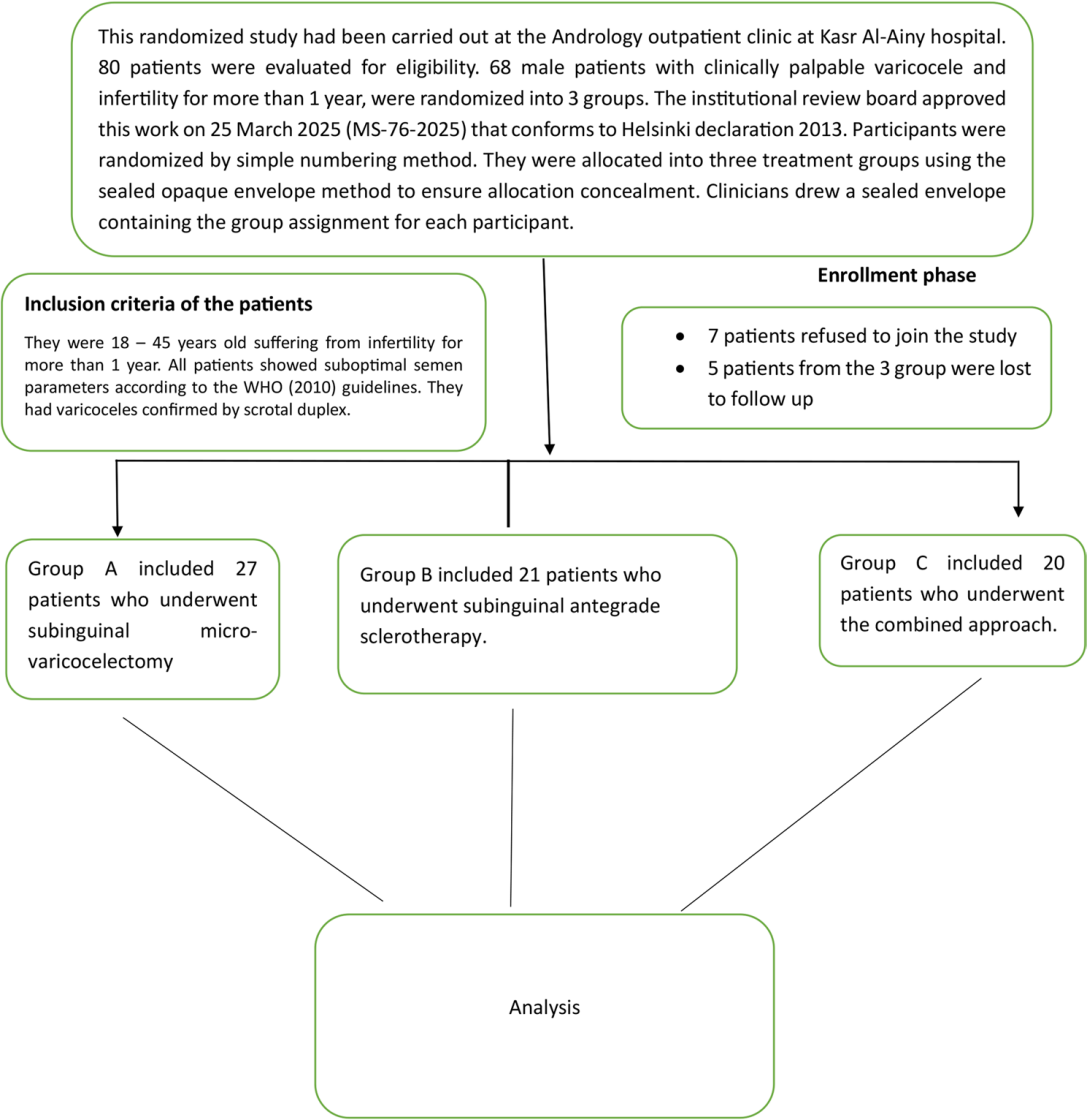
A flow chart shows the methodology of the study

The current randomized study had been carried out at the Andrology outpatient clinic at Kasr Al-Ainy hospital. 68 male patients with clinically palpable Vx and infertility for more than 1 year, were randomized into 3 groups (Fig. 1). The institutional review board approved this work on 25 March 2025 (MS-76-2025) that conforms to Helsinki declaration 2013 [12]. All patients were provided with detailed information about the three surgical techniques before the random grouping. Furthermore, they were informed about the potential differences in efficacy, operative time, and possible complications among the three techniques. All information was securely stored at the research site. All participants’ information was stored in locked file cabinets in areas with restricted access to the operating physicians. Participant’s research information wasn’t released outside the research center without his permission.

### Inclusion criteria of the patients

They were 18–45 years old suffering from infertility for more than 1 year. All patients showed suboptimal semen parameters according to the WHO (2010) guidelines [13]. They had Vx confirmed by scrotal duplex.

### Exclusion criteria of the patients

Patients with genito-urinary tract infections, erectile dysfunction, diminished sexual desire or inhibited male orgasm, uncontrolled systemic illnesses, alcoholics or drug abusers, patients with hormonal disturbance and patients with azoospermia were all excluded.

### All participants were subjected to the following

Past and surgical histories were obtained. General and local examinations were done. Participants were randomized by simple numbering method. They were allocated into three treatment groups using the sealed opaque envelope method to ensure allocation concealment. Clinicians drew a sealed envelope containing the assigned technique for each participant. Group A included 27 patients who underwent subinguinal MSV. Group B included 21 patients who underwent SAS. Group C included 20 patients who underwent a combined approach. This randomization process ensured equal distribution of participants across the groups, minimizing selection bias and supporting the study validity. Vx was graded according to Bertolotto et al. (2021) guidelines [14]. Routine laboratory investigations including complete blood count (CBC), blood group and Rh, coagulation profile, kidney function tests and blood glucose levels were requested for all patients. All participants brought 3 semen samples at the beginning of the study and after 3 months and 6 months, respectively.

The semen samples were analyzed according to the WHO (2010) guidelines [13]. Serum follicle stimulating hormone (FSH), luteinizing hormone (LH), and testosterone levels were evaluated using chemiluminescence immunoassay (CLIA) technique (1.5–14 mIU/ml for FSH, 1.5–8 mIU/ml for LH, and 2.4–8.3 ng/ml for total testosterone). All assays were executed utilizing Cobas E411 immunoassay analyzer (Roche Diagnostics GmbH, Mannheim, Germany). All patients were evaluated according to the guidelines set by the Scrotal and Penile Imaging Working Group of the European Society of Urogenital Radiology (ESUR-SPIWG) (2020) [15]. Patients were examined in the standing position, and throughout valsalva maneuver by the scrotal duplex in the department of andrology and STDs, Kasr Al-Ainy Faculty of Medicine (Versana Essential™ Ultrasound). Testicular Volume (in mL) was measured by the same duplex. The maximum diameter of the veins in the pampiniform plexus was determined. The presence and duration of reflux (reverse blood flow) during valsalva were documented. Reflux lasting > 2 s was significant. It should be noted that the scrotal duplex was repeated by the same assigned physician after 6 months using the same instructions to determine recurrence in the recruited cases.

### Techniques of different approaches

**Fig. 2:**
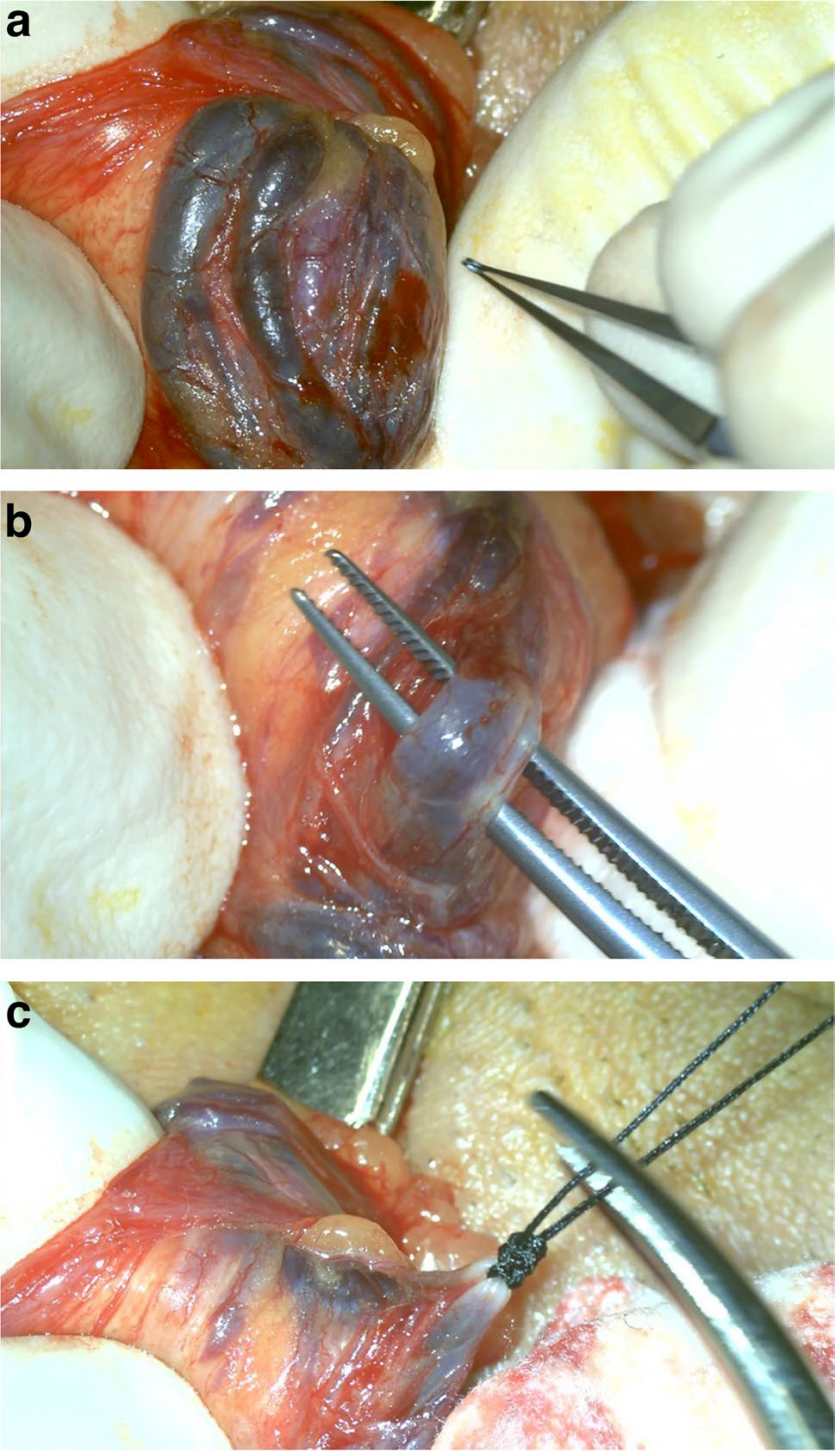
**a **shows the first step of delivering the spermatic veins. **b** shows the dissection of the spermatic veins using the microsurgical loupe. **c** shows the ligation of the spermatic veins using the microsurgical loupe

All groups were scheduled for subinguinal approach at the site of the external inguinal ring that was identified by invaginating the scrotal skin with an index finger parallel to the spermatic cord as it passed over the pubic tubercle. An oblique incision of 1.5 to 2.5 centimeters long, was made along Langer’s lines just under this level. Camper’s and Scarpa’s fasciae were subsequently separated with electrocautery. The wound was deepened by blunt dissection applying Army/Navy retractors until the level of the spermatic cord was reached. The cord was recognized as it passed over the pubic tubercle [Figure 2a].

Once identified, the cord was encircled by a Babcock instrument. A 1-inch Penrose drain was then looped underneath the cord and used to gently deliver the cord from the wound. The floor of the incision was inspected at this point to ensure the delivery and isolation of the entire cord.

### Patients in group A

Subinguinal MSV was adopted using a surgical microscope (Leica M 320, 6x to 25x, Germany) to allow sparing of the artery and lymphatics and ligation of all visible veins using 2 − 0 silk (Fig. 2a, b and c) [16].

### Patients in group B

**Fig. 3:**
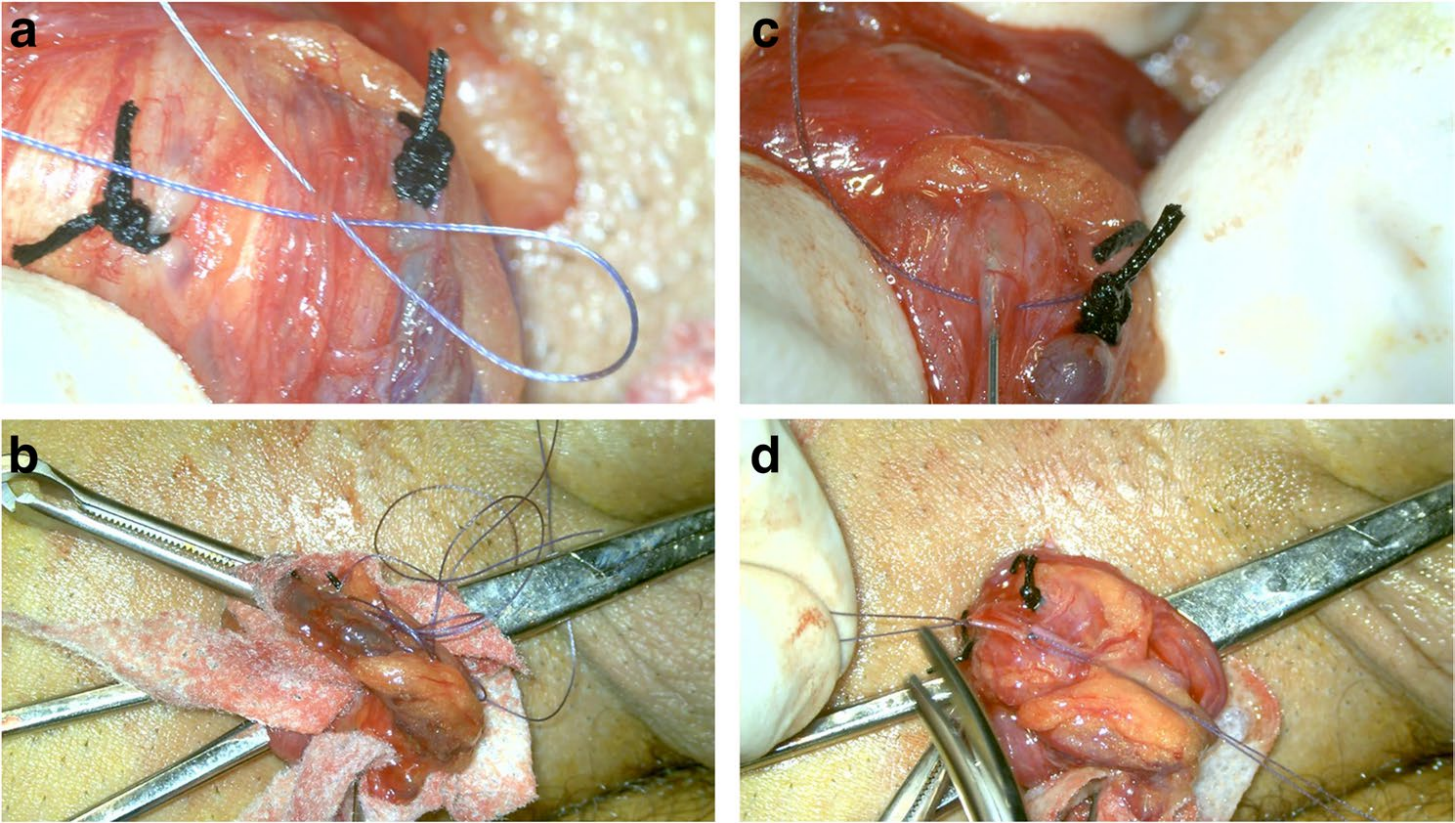
**a** shows the first step of identifying the target vein for sclerotherapy injection. **b** shows proximal and distal spermatic cord occlusion. **c** shows the injection of sclerosant into the target vein of the spermatic cord. **d** shows proximal and distal ligations of the target spermatic vein after injecting the sclerosant

SAS, with proximal and distal occlusion of the cord by bulldog clamps followed by direct injection of aetoxisclerol 3% through 30-gauge needle into the largest spermatic vein then proximal and distal ligation of the punctured vein by 2 − 0 silk followed by saline wash of the spermatic cord (Fig. 3a, b, c and d) [17].

### Patients in group C

The combined approach was adopted by dissection and ligation of the largest visible veins using the same surgical microscope accompanied by proximal and distal occlusion of the cord by bulldog clamps followed by distal ligation of one of the periarterial veins selected for injection of aetoxisclerol 3% through 30 gauge needle then proximal ligation of the punctured vein by 2 − 0 silk followed by saline wash of the spermatic cord (Fig. 3a, b, c and d) [18].

### Sample size determination

The required sample size was calculated using G*Power statistical package program Version 3.1.9.7 (Franz Faul, Universitat Kiel, Germany). The following assumptions: confidence level=95%; power (1-β): 95%; effect size=0.25 (ideal); number of groups = 3; number of measurements =3 were utilized using F test (repeated measures MANOVA test, between factors) for this randomized controlled clinical trial study design. Considering 10% non-response rate, the total sample size was 60 patients classified into 20 patients in each arm. A repeated measures effect size (W2) could be derived from the F ratio, the numbers of repetitions (K), and the sample size (n) W2 = (K — 1)(F — 1)/(K — 1)(F — 1)nk [19]. Calculation of (N) for multiple means was done using this equation N = 𝜆/Cohen f ^2^ [19]. In a repeated measures study, the correlation between the measures has a dramatic effect on the power of the test, and this effect increases with the number of measurements [19]. Different authors appear to differently define the effect size (f) differently [19]. Some of them define it as follows σ(m)/σ [19]. Others define it as follows σ(m)/σ*C [19]. Where c is based on the correlation between the measures C= √ K/(1−ρ) [19]. So, when f=0.25 in the power analysis, this is sometimes assuming a correlation of 0 [19].

### Statistical analysis

Data had been analyzed utilizing SPSS version 25. Continuous variables (e.g., sperm count, motility, morphology) had been represented as mean ± SD, & categorical variables (e.g., complication & recurrence rates) as frequencies & percentages.

Baseline characteristics were compared across groups using one-way ANOVA for continuous variables and the chi-square test for categorical ones. Changes in semen parameters over time (baseline, 3 months, 6 months) were assessed using repeated determines ANOVA. Group variances had been examined with one-way ANOVA for continuous outcomes and post-hoc Tukey’s test for pairwise comparisons, while categorical results used the Fisher’s exact test or chi-square. Effect sizes (e.g., partial eta-squared) and 95% confidence intervals were reported. Assumptions of normality and variance homogeneity were tested. *P* value of under 0.05 indicated significant outcomes.

## Results

Table 1 showed that the mean age was higher in group (C) but the difference was not significant. Regarding Vx side, bilateral varicocele was more frequent than left-sided in all groups, with no significant difference between them (Table 1). Moreover, primary infertility was more frequent in group (A) (Table 1). While secondary infertility was more frequent in groups B and C (Table 1). Distribution of grades II and III Vx on the left side was roughly similar among the three groups, 51.9%, 52.4%, 55%, 48.1%, 47.6%, & 45%, respectively (Table 1). Distribution of grades I and II Vx on the right side had been noticed as follows 43.5%, 25%, 35.3%, 56.5%, 75%, 64.7%, respectively (Table 1). Furthermore, left testicular volume was preoperatively and postoperatively comparable among the three groups (11.86 ± 2.75, 12.23 ± 2.06, 12.12 ± 2.3, 11.94 ± 2.84, 11.93 ± 1.94, 11.92 ± 2.04, respectively) (Table 1).

**Table 1:** shows age and clinical data of the participants

		Group A (*N* = 27)	Group B (*N* = 21)	Group C (*N* = 20)	Test	*P* value
Age	Mean	31.4	31.1	34.05	F = 1.366	0.262
	±SD	± 6.04	± 6.5	± 6.12		
Side of varicocele	Left	4 (14.8%)	5 (23.8%)	3 (15%)	X^2^=0.794	0.67
	Bilateral	23 (85.2%)	16 (76.2%)	17 (85%)		
Type of infertility	1ry infertility	23 (85.2%)	14 (66.7%)	12 (60%)	X^2^=4.058	0.131
	2ry infertility	4 (14.8%)	7 (33.3%)	8 (40%)		
Grade of left varicocele	Grade 2	14 (51.9%)	11 (52.4%)	11 (55%)	X^2^=0.05	0.97
	Grade 3	13 (48.1%)	10 (47.6%)	9 (45%)		
Grade of right varicocele	Grade 2	14 (51.9%)	11 (52.4%)	11 (55%)	X^2^=1.405	0.49
	Grade 3	13 (48.1%)	10 (47.6%)	9 (45%)		
Left testicular volume	Preoperative	11.86 ± 2.75	12.23 ± 2.06	12.12 ± 2.3	F = 0.147	0.86
	Postoperative	11.94 ± 2.84	11.93 ± 1.94	11.92 ± 2.04	F = 0.0004	0.99
right testicular volume	Preoperative	13.95 ± 2.55	13.52 ± 1.96	14.05 ± 2.02	F = 0.3372	0.71
	Postoperative	13.94 ± 2.62	12.97 ± 2.08	13.94 ± 1.94	F = 1.320	0.27
Operative time for left varicocele in minutes	median	29.5	20	25	H = 5.58	0.06
	range	3–40	19–20	20–26		
Operative time for bilateral varicoceles in minutes	Mean	57.86	36.93	44.17	F = 110.7679	≤ 0.001
	±SD	± 5.2	± 4.3	± 3.45		

Similarly, mean pre-operative and postoperative right testicular volumes did not differ significantly among all groups (13.95 ± 2.55, 13.52 ± 1.96, & 14.05 ± 2.02,13.94 ± 2.62, 12.97 ± 2.08, 13.94 ± 1.94, respectively) (Table 1). Moreover, the median operative time for left-sided procedures in all groups was 29.5 min (range 3–40), 20 min (range 19–20), 25 min (range 20–26), respectively (Table 1). Regarding bilateral procedures, the mean operative time was significantly longer in group (A) (57.86 ± 5.2 min) compared to groups B and C (36.93 ± 4.3 min; 44.17 ± 3.45 min, respectively) (Table 1). There was insignificant difference in the pre-operative mean left testicular vein diameter (Table 2). Meanwhile, a highly significant reduction in the post-operative mean left testicular vein diameter was observed among all groups, with the smallest vein diameter being observed in group B (Table 2). Similarly, pre-operative left vein reflux was comparable among all groups (Table 2). Additionally, post-operative left vein reflux demonstrated insignificant difference among all groups (Table 2). There was insignificant difference among all groups in pre-operative and post-operative right vein diameter (Table 2). Right reflux was preoperatively present in all cases and postoperatively absent in all cases (Table 2). A highly significant post-operative reduction in the left vein diameter was noted in the intra-group comparisons (Table 2), as well as significant non visualization of the post-operative left vein reflux (Table 2). Similarly, post-operative right vein diameter significantly reduced in the intra-group comparisons (Table 2), as well as significant non visualization of the post-operative right vein reflux (Table 2). The median count significantly increased at baseline, and after 3 months, and 6 months, respectively (Table 3). Intra-groups comparison showed significant improvement over time in group (A), group (B), and group (C) (Table 3).

**Table 2:** shows scrotal duplex findings before and after different interventions among the participants

	Variable			Group A (*N* = 27)	Group B (*N* = 21)	Group C (*N* = 20)	*P* value
SC duplex – Lt diameter (mm)	Preoperative		Mean	4.10	4.09	4.09	0.99
			±SD	± 0.98	± 0.57	± 0.94	
	Postoperative		Mean	2.85	1.78	2.1	≤ 0.001
			±SD	± 0.55	± 0.19	± 0.25	
	*P* value			≤ 0.001	≤ 0.001	≤ 0.001	
Lt regurge preoperative	Positive			27 (100%)	21 (100%)	20 (100%)	1
	Negative			0 (0%)	0 (0%)	0 (0%)	
Lt regurge post operative	Positive			1 (3.7%)	0 (0%)	0 (0%)	0.46
	Negative			26 (96.3%)	21 (100%)	20 (100%)	
	*P* value			≤ 0.001	≤ 0.001		
SC duplex – Rt diameter (mm)	Preoperative	Mean		2.86	2.97	2.81	0.54
		±SD		± 0.55	± 0.43	± 0.40	
	Postoperative	Mean		2.1	1.89	1.94	0.07
		±SD		± 0.36	± 0.37	± 0.21	
	*P* value			≤ 0.001	≤ 0.001	≤ 0.001	
Rt regurge preoperative				*N* = 23	*N* = 16	*N* = 17	
	Positive			23 (100%)	16 (100%)	17 (100%)	1
	Negative			0 (0%)	0 (0%)	0 (0%)	
Rt regurge post operative	Positive			0 (0%)	0 (0%)	0 (0%)	1
	Negative			23 (100%)	16 (100%)	17 (100%)	
	*P* value			≤ 0.001	≤ 0.001	≤ 0.001	

**Table 3:** shows changes in semen parameters among the participants at baseline and after 3 months and 6 months

		Group A *N* = 27	Group B *N* = 21	Group C *N* = 20	*P* value
Sperm count in mil/ml	Baseline	10.0 (0.3–41.0)	13.0 (2.0–68.0)	20.0 (0.1–150.0)	0.189
	3 months	30.0 (1.0–100.0)	36.0 (5.0–100.0)	50.0 (1.0–250.0)	0.3
	6 months	50.0 (3.0–120.0)	40.0 (6.0–70.0)	80.0 (3.0–300.0)	0.148
	*P* value	≤ 0.001	0.005	0.03	
Sperm total motility	Baseline	30.0 (0.0–70.0)	36.0 (5.0–60.0)	39.0 (0.0–80.0)	0.748
	3 months	50.0 (0.0–70.0)	45.0 (20.0–60.0)	50.0 (10.0–70.0)	0.687
	6 months	60.0 (10.0–70.0)	55.0 (30.0–70.0)	60.0 (20.0–70.0)	0.556
	*P* value	0.03	0.001	0.002	
Sperm progressive motility	Baseline	10.0 (0.0–55.0)	5.0 (0.0–30.0)	10.0 (0.0–50.0)	0.405
	3 months	25.0 (0.0–60.0)	20.0 (0.0–45.0)	25.0 (0.0–65.0)	0.187
	6 months	30.0 (0.0–60.0)	25.0 (0.0–50.0)	37.5 (5.0–65.0)	0.197
	*P* value	≤ 0.001	≤ 0.001	≤ 0.001	
Sperm abnormal forms	Baseline	95.0 (20.0–100.0)	65.0 (35.0–100.0)	95.5 (45.0–100.0)	0.003
	3 months	80.0 (35.0–98.0)	60.0 (50.0–90.0)	90.0 (60.0–90.0)	≤ 0.001
	6 months	80.0 (20.0–95.0)	55.0 (50.0–65.0)	65.0 (35.0–90.0)	0.003
	*P* value	0.004	0.03	≤ 0.001	

The median total sperm motility insignificantly increased among the participants at baseline, and after 3 months, and 6 months, respectively (Table 3). However, intra-groups comparison showed statistically significant improvement regarding the median total sperm motility among the participants over the same period (Table 3). Similarly, the median progressive motility did not show any significant improvement among the participants at baseline, after 3 months and 6 months, respectively (Table 3). However, intra-groups comparison showed significant improvement in the median progressive motility among the participants over the same period (Table 3). Remarkably, the median abnormal forms significantly reduced between the 3 groups at baseline, after 3 months and 6 months, respectively (Table 3). Consistently, the intra-groups comparison among the participants revealed significant reductions in the abnormal forms over the same period (Table 3). It should be mentioned that insignificant pregnancy rate was noted among the groups that was as follows 22.2%, 19%, and 25%, respectively. The frequency of complications among the participants was demonstrated in Table 4. Binary logistic regression analysis for prediction of pregnancy after 6 months demonstrated that a 6-months progressive sperm motility was identified as a significant predictor of pregnancy (OR = 1.039, 95% CI: 1.000–1.079, *p* = 0.05). Other factors including age, infertility type, baseline and 3-months semen parameters (volume, count, total and progressive motility), and clinically graded left and right varicoceles, were not statistically significant (all *p* > 0.05). Remarkably, baseline total sperm motility (OR = 1.028, *p* = 0.073) and baseline volume (OR = 0.579, *p* = 0.094) showed borderline trends but did not reach statistical significance.

**Table 4:** shows the frequency of complications between the participants

	Group A*N* = 27	Group B*N* = 21	Group C*N* = 20	*P* value
Hydrocele	1 (3.7%)	0 (0%)	0 (0%)	0.46
Orchialgia	1 (3.7%)	4 (19%)	0 (0%)	0.04
Recurrence	1 (3.7%)	0 (0%)	0 (0%)	0.46
Scrotal edema	0 (0%)	1 (4.8%)	0 (0%)	0.32
Scrotal swelling and mild bluish discoloration	0 (0%)	0 (0%)	2 (10%)	0.08

## Discussion

Our results showed insignificant difference in the mean age of the participants. This could be seen in alignment with Ahmed et al. (2008) [20] and Cannarella et al. (2024) [7] and Elahwany et al. (2025) [11]. We found that the distribution of clinical grades of Vx on the left side was roughly similar among all groups. Grade I Vx had been detected in the current study on the right side as follows, 43.5%, 25%, and 35.3%, respectively. While grade II Vx had been detected on the right side as follows, 56.5%, 75%, and 64.7%, respectively. In contrast, Botha & Heyns (2006) [21] and Ahmed et al. (2008) [20] reported that most cases presented in their study were grade III Vx. In the present study, the mean left and right testicular volumes were preoperatively and postoperatively comparable among all groups. Moreover, insignificant difference was preoperatively observed in the mean left testicular vein diameter. While postoperatively, a highly significant reduction in the left vein diameter was observed with the smallest vein diameter being observed in group B. Moreover, the left vein reflux was preoperatively and postoperatively comparable. Intra-group comparisons showed a highly significant reduction in the left vein diameter postoperatively together with invisibility of the left vein reflux. There was insignificant difference among all groups in the right vein diameter preoperatively and postoperatively. The right vein reflux was preoperatively seen in all cases and postoperatively invisible in all cases. Consistently, intra-group comparisons demonstrated significant reduction in the right vein diameter as well as significant invisibility of the right vein reflux postoperatively. Our results agreed with Cannarella et al. (2024) [7] who stated that there was no significant difference among all groups regarding the mean left and right testicular volumes.

In contrast, Elahwany et al. (2025) [11] revealed significant difference in the left testicular volume in their cases, being largest in the group who underwent Fisch technique. Additionally, the same study demonstrated significant reduction in the right and left veins diameters especially in the group who underwent sclerotherapy. Our current study showed that the median operative time for the left-sided procedures in all groups were as follows 29.5 min, 20 min, and 25 min, respectively. Consistently, Colpi et al. (2006) [17] reported that the mean operative time was 25 min for SAS. Regarding bilateral procedures, the mean operative time was significantly prolonged especially in group A. In a similar trend, Corvin et al. (2001) [18] and Botha & Heyns (2006) [21] and Ahmed et al. (2008) [20] reported similar findings. Our current study showed that the median count significantly increased within each group at baseline and after 3months and 6 months, respectively. In conjunction with our findings, Fayez et al. (2010) [22] stated that SAS and the inguinal surgery (Ivanissevich technique) groups were associated with a significant rise in the mean sperm count during the follow-up visits compared to the patients who underwent scrotal antegrade sclerotherapy (Tauber’s technique). Similarly, Cannarella et al. (2024) [7] demonstrated a significant rise in the sperm concentration in cases who underwent sclerotherapy that was conducted through the right common femoral vein or basilic vein. On the other hand, Botha & Heyns (2006) [21] and Ahmed et al. (2008) [20] reported an insignificant difference among both methods regarding improvement in the mean sperm count. Our study demonstrated a significant improvement in the mean total sperm motility in all patients. Similarly, Fayez et al. (2010) [22] reported that SAS and the inguinal surgery (Ivanissevich technique) groups were associated with a significant improvement in the mean total motility during the follow-up visits compared to the patients who underwent scrotal antegrade sclerotherapy (Tauber’s technique).

Consistently, a meta-analysis conducted by Wang et al. (2015) [23] reported that inguinal and subinguinal MSV were associated with significant improvement in the sperm count and motility. In the same context, Cannarella et al. (2024) [7] reported significant difference between the studied groups regarding total sperm motility. Our results also agreed with Ahmed et al. (2008) [20] and Cannarella et al. (2024) [7] who stated that there was a significant improvement regarding progressive motility among their patients. In the present study, the median abnormal forms significantly reduced during the follow up period. Additionally, intra-group comparisons revealed significant reductions during the same follow-up period. Our results agreed with Botha & Heyns (2006) [21] who reported a significant reduction in the sperms abnormal morphology from baseline to twelve months in their patients. On the other hand, Ahmed et al. (2008) [20] and Fayez et al. (2010) [22] and Cannarella et al. (2024) [7] reported insignificant difference among their patients regarding teratozoospermia. Pregnancy rate in the current study occurred in the 3 groups as follows 22.2%, 19%, and 25%, respectively. However, this rate was insignificant. This insignificant finding may be explained by the short duration of the follow-up of the patients in the current study. Consistently, Fayez et al. (2010) [22] reported 12.24% pregnancy rate in the SAS group. In contrast, Botha & Heyns (2006) [21] reported 50% pregnancy rate in the ASS group who were 6 patients and the same rate in the inguinal micro-varicocelectomy (IMV) group who were 8 patients after one year of their respective surgeries. Furthermore, a meta-analysis conducted by Wang et al. (2015) [23] reported that inguinal & subinguinal MSV had the highest pregnancy rates. In view of the above-mentioned facts, it should be mentioned that subinguinal MSV is the best approach for male infertility [22] and SAS is also an effective approach for male infertility [11].

Henceforth, the combined approach is recommended because of a relatively short operative time that was noted for the bilateral procedure because of ligating lesser number of veins using the MSV as the rest of the veins would be occluded by the sclerosant agent [18]. Moreover, it is associated with the lowest incidence of complications. Regarding complications among the patients, our results showed that hydrocele occurred in 3.7% of group A, with insignificant difference among all groups. Orchialgia was significantly more frequent in group B (19%) compared to 3.7% in group A and none in group C. This interesting finding could be explained by the fact that the combined approach required lesser amount of the sclerosant agent. However, the role of the postoperative time in lessening the severity of pain could not be excluded as the patients were followed up for 6 months only post-operative. Recurrence rate was only reported in group A (3.7%) and scrotal edema was only reported in group B (4.8%). Also, these observations were insignificant. Scrotal swelling with mild bluish discoloration was insignificantly noted in 10% of group C. Our results are consistent with Corvin et al. (2001) [18] and Colpi et al. (2006) [17] and Fayez et al. (2010) [22], who reported that hydrocele was never experienced in the sclerotherapy group. In addition, Cannarella et al. (2024) [7] demonstrated insignificant difference among the patients regarding hydrocele rate. In the same context, Ahmed et al. (2008) [20] reported insignificant differences in terms of early or late postoperative complications in their procedures. Furthermore, Botha & Heyns (2006) reported one patient (8%) who had a cord hematoma in the ASS group together with no complications recorded in the IMV group.

Consistently, Wang et al. (2015) [23] indicated that Tauber antegrade sclerotherapy and subinguinal MSV were associated with the lowest risk of hydrocele. Thus, it should be mentioned that SAS can be a safe and effective method in treating male infertility especially when combined with subinguinal MSV. However, 2 case reports reported serious complications following scrotal antegrade sclerotherapy including large bowel infarction and spinal cord injury [24, 25].

### Limits of the study

There are several limitations of the current study that should be stated. Firstly, the sample size was relatively small. Secondly, the patients were followed up for a short duration that might explain the insignificant pregnancy rate that was reported in the study. Furthermore, the short period of follow-up could not properly assess the role of timing in lessening the severity of the testicular pain that was mainly reported in the SAS group. Nevertheless, Ahmed et al. (2008) stated that timing did not play any role in the incidence of the complications that were reported in their study as they did not find any significant difference between early and late complications among their patients [20]. Additionally, exclusion of cases with hormonal imbalance deprived us from evaluating the role of the reproductive hormones from predicting pregnancy occurrence in the study. However, there were 5 cases enrolled with hormonal imbalance. Finally, the 6th edition of the WHO guidelines for semen analysis was not utilized for interpretation could be added as another limitation of the study [26]. Nevertheless, it should be mentioned that the 5th edition is more simplified and a user-friendly guide regarding different conventional and extended semen tests [13].

In the same context, several concerns were raised about the 6th Edition of the WHO guidelines for semen analysis interpretation that should be explained for male infertility specialists [27].

## Conclusion

Subinguinal MSV and SAS resulted in significant postoperative improvements in semen parameters. Nevertheless, combined approach should be recommended as it is associated with the lowest complications incidence. Future studies with larger scales are needed to affirm the current results.

## Data Availability

The data that support the findings of this study are available from the corresponding author upon reasonable request.

## Abbreviations

MSV: microsurgical varicocelectomy
SAS: subinguinal antegrade sclerotherapy
CLIA: Chemiluminescence immunoassay
FSH: Follicle stimulating hormone
LH: Leutinizing hormone
Vx: varicocele
